# Ab-initio evaluation of acid influence on chemical stability of hydrophilic diglycolamides

**DOI:** 10.3389/fmolb.2022.1063022

**Published:** 2022-12-07

**Authors:** Jakub Luštinec, Tomáš Koubský, Ladislav Kalvoda

**Affiliations:** Department of Solid State Engineering, Faculty of Nuclear Sciences and Physical Engineering, Czech Technical University in Prague, Prague, Czechia

**Keywords:** diglycolamides, hydrophilic DGA, density functional theory, radiolytic stability, acid influence

## Abstract

Diglycolamides (DGA) form one of the most promising groups of organic ligands used in bio-inspired solvent extraction processes of lanthanide and actinide ions. Continuous experimental and theoretical research is still performed in order to further improve their application properties including their chemical stability in the real extraction environment. This work provides results of our theoretical approach focused on inclusion of an acid influence on the DGAs chemical structure, treated in frame of the density functional theory. Three different models describing the acid action are proposed and investigated in attempt to increase the resulting accuracy of the chemical stability predictions based on verified theoretical descriptors. The procedure is applied and tested on the set of selected hydrophilic DGA representatives. Comparison of the model results obtained with and without acid action shows that two types of protection effects may occur: a ‘direct’ protection, accompanied by an explicit change of the ligand stability indicators, and an ‘indirect’ one consisting in reaction of acid molecules with radicals preceding the contact of latter with the extracting ligands. The possibility of the direct acid protection route is supported by the significant decrease of the Fukui charges found with the acid models included. On the other hand, there is in general no significant difference of trends in the calculated chemical stability descriptors suggesting that an indirect mechanism must be also considered in order to explain the experimentally observed protective role of acids on the chemical stability of investigated DGA derivatives.

## Introduction

Closing the nuclear fuel cycle and the maximal usage of uranium contained in the nuclear fuel are one of the biggest recent challenges related to a sustainable nuclear power plants operation. The spent nuclear fuel contains fission products, unused U, and minor actinides (MAs) which are, together with Pu, also responsible for the long term radiotoxicity of the nuclear waste. One of the possibilities for the spent nuclear fuel treatment is its partitioning and transmutation (Veliscek-Carolan, 2016). Partitioning consists of co-extraction of MAs and lanthanides from a liquid solution of nuclear waste and the subsequent separation of these components from each other. After partitioning, the transmutation of MAs is performed in order to reduce their long radiative decay lifetime and toxicity. In the result, the radiotoxicity of MAs and remaining waste can be significantly reduced (Veliscek-Carolan, 2016).

Among other organic molecules used in solvent extraction procedures, one of the most promising groups is formed by diglycolamides (DGAs). DGAs are especially well applicable for extraction of trivalent actinide and lanthanide ions, mimicking thus some of the processes observed in biological systems (Mattocks and Cotruvo, 2020). The solubility of DGAs is determined by the optional presence and length of specific N-alkyl substituents. The DGA derivatives containing short N-alkyls are water soluble, e.g., tertamethyldiglycolamide (TMDGA) or tetraethyldiglycolamide (TEDGA). These hydrophilic molecules are used as aqueous stripping and back-holding agents (Sasaki et al., 2007) in ALSEP process (Actinide Lanthanide Separation) (Lumetta et al., 2014) or EXAm process (Extraction of Americium) (Rostaing et al., 2012).

**FIGURE 1:**
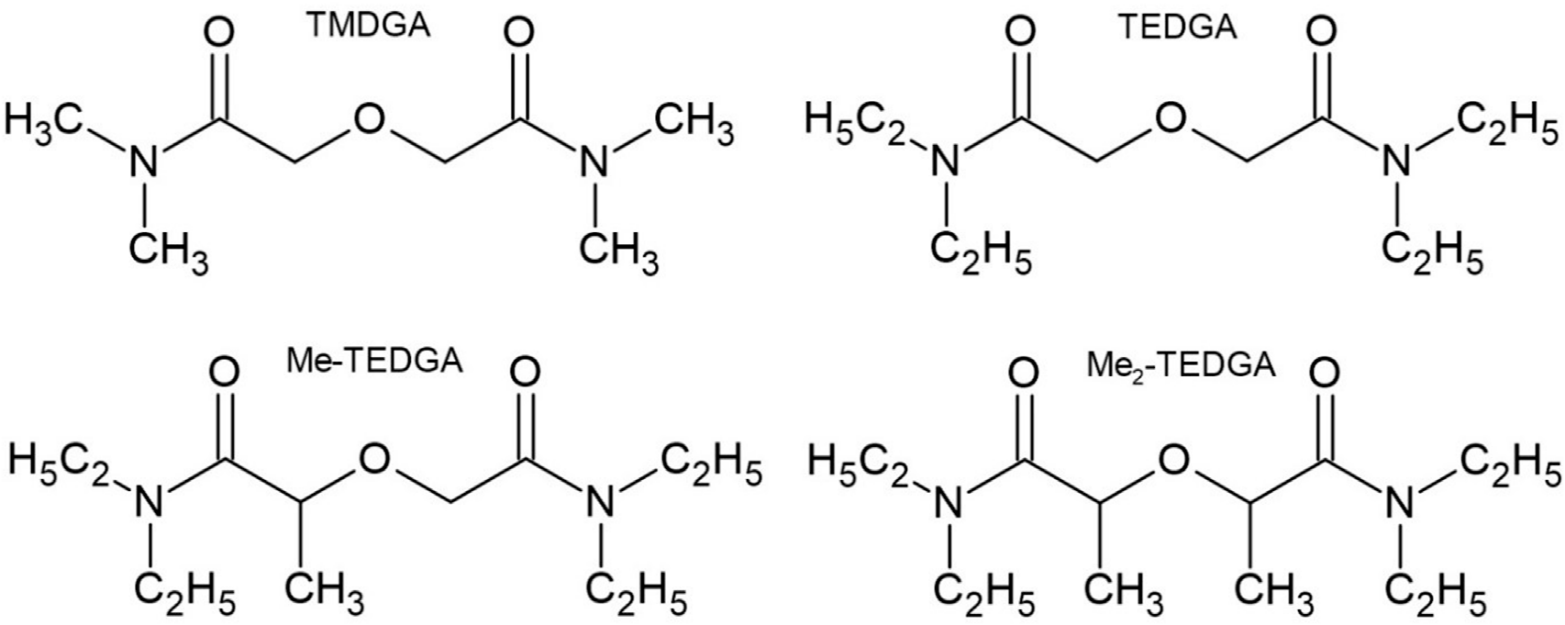
Studied hydrophilic DGA derivatives.

In this work, we study the following DGAs representatives (Figure 1).

• TMDGA (N,N,N′,N′-tetramethyl-diglycolamide; 2,2'_oxybis (N,N-dimethylacetamide))
• TEDGA (N,N,N′,N′-tetraethyl-diglycolamide; 2,2′-oxybis (N,N-diethylacetamide))
• Me-TEDGA (2-(2-(diethylamino)-2-oxoethoxy)-N,N-diethylpropanamide)
• Me_2_-TEDGA (2,2′-oxybis (N,N-diethylpropanamide)).

As indicated by experimental results (Wilden et al., 2018; Horne et al., 2019), addition of methyl group in Me-TEDGA and Me_2_-TEDGA results in remarkable radiolytic stability enhancement compared to the un-methylated TEDGA.

In our previous work (Koubský and Luštinec, 2018), the quantum mechanical indicators of radiolytic stability of the four above mentioned hydrophilic DGA derivatives were already evaluated and analysed in the environment of pure water. Especially, radical Fukui function, Fukui charges (condensed Fukui functions), and bond orders were found to be of key importance for the related radiolytic stability predictions. This work extends the theoretical treatment on the problem of acid influence implementation into the DFT calculations in order to improve the stability predictions. In particular, above mentioned verified stability indicators are calculated using three different acid inclusion models, and the obtained results compared and juxtaposed with the former acid-free results (Koubský and Luštinec, 2018). In addition, the condensed dual descriptor (CDD) $∆f^{A}$ proposed by (Morell et al., 2005; Morell et al., 2006) and applied by Smirnova et al. (2020) for the radiolytic stability predictions of several extractants is evaluated and discussed.

### Reference experimental studies

Two key experimental radiolytic stability studies of the hydrophilic DGA derivatives shown in Figure 1 were performed by Wilden et al. (2018) and Horne et al. (2019).

In the first study, Wilden and co-workers tested solutions of DGA derivatives in a warmed nitric acid environment and compared the results with the behaviour in a pure water environment. In addition to the steady state measurements, the pulsed radiolysis method followed by kinetics measurements and mass spectroscopy was also applied, in order to get a deeper insight into the radiolysis process of the hydrophilic DGA derivatives. High rate constants for DGAs reactions with the hydroxyl radical are found, suggesting the important role of this radical in the radiolytic degradation mechanism in water environment. Observed decrease in dose constants with the increasing molecular weight of DGAs suggests, together with the measured rate constants, an electron transfer as the mechanism of the radical reaction. The radiolytic stability found for studied molecules follows the trend TMDGA < TEDGA < Me-TEDGA < Me_2_-TEDGA, i.e., growths with the molecular weight of the tested derivative (Wilden et al., 2018).

The second experimental study performed by Horne et al. (2019) (using the setup similar to the setup used by Wilden and co-workers) deals with a neutral pH concentrated aqueous nitrate solutions of the four selected hydrophilic DGA derivatives. The authors conclude that the studied hydrophilic DGAs undergo a first-order decay; the observed degradation product distributions are similar to those found in Wilden et al. (2018) under pure water conditions (except for the additional appearance of NOx adducts), and the radiolysis is driven by hydroxyl and nitrate radical oxidation chemistry, the latter then likely moderated by some secondary reactions scavenging the degradation products (Horne et al., 2019). The radiolysis rate of hydrophilic DGA representatives in aqueous nitrate solutions is found to be significantly reduced and less structurally sensitive compared to the acid-free solutions, similarly to the situation already observed for lipophilic DGA derivatives (Galan et al., 2015).

### Degradation reaction mechanisms

As in our previous work (Koubský and Luštinec, 2018), we generally consider indirect radiolysis mechanisms to prevail, because of the low actual concentration of ligands used in extraction solutions (Wilden et al., 2018). Such indirect process consists in the primary radiolysis of solvent molecules, followed then by reactions of the radiolysis products with the ligand molecules. These water radiolysis products are represented by OH^•^ and H^•^ radicals. The overall reaction conditions anticipated in this work follow the experiments performed by Wilden et al. (2018), adding to the pure water environment [considered in the previous study (Koubský and Luštinec, 2018)] the influence of nitric acid *via* the proposed acid models.

The first degradation reaction mechanism considered here is supposed to start with the hydrogen abstraction followed by rupture of the ether bond C-O, in analogy with the lipophilic DGAs (Koubský et al., 2017). In the previous works (Koubský et al., 2017; Koubský and Luštinec, 2018), it was concluded that the hydrogen abstraction is more probable to occur on the ether group than on the side chains. Therefore, the ether group is mainly investigated in this work. It is worth to notice that the methylation of the ether carbons that promotes the higher molecular stability, lowers also the number of ether hydrogens: four in case of the two non-methylated studied DGA derivatives TMDGA and TEDGA, three in case of Me-TEDGA, and two for Me_2_-TEDGA.

The second possible degradation reaction mechanism follows the findings of Wilden et al. (2018). This mechanism is based on the known oxidation nature of the hydroxyl radical OH^•^ that could cause the electron transfer from the amide group producing the DGA radical cation [DGA]^•+^. Afterwards, the rupture of the ether C-O bonds or the amide C-N bonds occurs (Wilden et al., 2018).

## Methods and computational settings

### Applied acid models

The acid influence is implemented into DFT simulations of the selected hydrophilic DGA derivatives by setting up three different testing models. In the first two of them, the interaction of ligand with dissociated acid molecules is assumed. These two models are independent of the particular acid type used in the experiments. In the first, simplest model, the hydrogen cation H^+^ is added to the calculated system in order to create a complex with the extraction molecule. In the second tested model, H_3_O^+^ cation (also applied in (Matveev et al., 2017)) is included in calculations instead of H^+^. Finally, in the third model, the interaction with undissociated HNO_3_ molecule is tested. The latter model is also relevant since the DGAs extractants are commonly dissolved in nitric acid solutions concentrated enough to contain a significant amount of undissociated HNO_3_ molecules (Wilden et al., 2018). This model explicitly includes the specific acid, in contrary to the first two models using general acid representations. The particular issue complicating mutual comparison of the results obtained by these models follows from the different total charge of the studied systems: it is equal to +1 for the first two models, and to zero for the last one. Thus, the behaviour of valence electrons and the related stability descriptors are affected by this difference.

### Calculated stability indicators

The Frontier orbital theory of Fukui (1982) relates the molecule reactivity to the charge density $\rho \left(r\right)$ with respect to electrophilic and nucleophilic properties of the reaction. Further developed by Parr and Yang (1989), Fukui functions are practical tool for qualitatively measuring and displaying the reactive regions of molecules. Fukui functions describe the sensitivity of charge density to losing or gaining electrons as follows

$$f^{+}\left(r\right)=\frac{1}{∆N}\left(\rho_{N+∆}\left(r\right)-\rho_{N}\left(r\right)\right)$$

$$f^{-}\left(r\right)=\frac{1}{∆N}\left(\rho_{N}\left(r\right)-\rho_{N-∆}\left(r\right)\right).$$

Where $f^{+}\left(r\right)$ and $f^{-}\left(r\right)$ is respectively nucleophilic and electrophilic Fukui function, and ∆N is a change in the number of electrons. Radical Fukui function $f^{0}\left(r\right)$ is then obtained as the average of the nucleophilic and electrophilic Fukui function.

Another possible implementation of the Fukui theory is the condensed Fukui function giving Fukui charges. Fukui charges are calculated from the atomic charges $q_{A}$ as follows

$$f_{A}^{+}=q_{A}^{anion}-q_{A}$$

$$f_{A}^{-}=q_{A}-q_{A}^{cation}$$

Here $f_{A}^{+}$ and $f_{A}^{-}$ is respectively the nucleophilic and electrophilic Fukui charge on the atom A. Radical Fukui charge $f_{A}^{0}$ on atom A is then given as the average of nucleophilic and electrophilic Fukui charges.

$$f_{A}^{0}=\frac{1}{2}(f_{A}^{+}+f_{A}^{-})$$

The CDD (Morell et al., 2005; Morell et al., 2006) for atom A is defined as follows

$$∆f_{A}=f_{A}^{+}-f_{A}^{-}$$

The sign of CDD indicates the vulnerability of the atomic site to the particular type of radical attack: the negative sign relates to electrophilic attack, the positive sign then to nucleophilic one.

Wiberg bond indices $W_{AB}$ (Wiberg, 1968) are calculated from the electronic overlap between two atoms, *A* and *B,* as follows

$$W_{AB}=\underset{\mu \in A}{\sum }\underset{\sigma \in B}{\sum }P_{\mu \sigma }^{2}$$

Here μ and σ is atomic orbital on atom *A* and *B,* respectively, and $P_{\mu \sigma }$ is the corresponding density matrix element.

### Computational settings

The DFT calculations were performed with DMol^3^ module from Materials Studio 8.0 (Delley, 1990; Delley, 2000) and Gaussian09 code (Frisch et al., 2013). The conformation analysis of the models combining extractants with the selected acid representation was performed using Gaussian09 code; the initial optimised extractants conformations were taken from the previous work (Koubský and Luštinec, 2018) where the geometry optimization was performed firstly with BLYP and subsequently with B3LYP functional.

The H^+^ and H_3_O^+^ cations were then added in vicinity of the atoms possessing negative partial charge, as calculated in Koubský and Luštinec (2018). In the case of the third model employing the undissociated nitric acid molecule, eight different initial conformations were generated for each of the tested extractants, with the HNO_3_ molecule placed gradually into eight different positions in vicinity of the carbonyl oxygens, ether oxygen and the two amide nitrogen atoms, which are all likely to create hydrogen bonds with the HNO_3_ molecule.

Geometry optimization of the initial conformations was performed using Gaussian09 code with the following settings: 6–31G (d,p) basis set (Petersson et al., 1988; Petersson and Al-Laham, 1991), PCM solvent model (Miertuš et al., 1981; Tomasi et al., 2005) with water taken as the solvent, GD3BJ dispersion correction (Grimme et al., 2010; Grimme et al., 2011), and B3LYP exchange and correlation functional (Beck, 1993). Gaussian09 code with NBO 6.0 (Glendening et al., 2013) was then used for the Natural population analysis and the Wiberg bond indices (Wiberg, 1968) calculations (the latter providing bond orders discussed in Section 3.4) with the same settings as the ones used in the conformation analysis.

Fukui functions, Fukui charges, and CDDs were calculated with DMol^3^ code using the following settings: DNP basis set (Delley, 1990), COSMO solvent model (Klamt and Schüürmann, 1993; Tomasi and Persico, 1994), GD2 dispersion correction (Grimme, 2006), and B3LYP exchange and correlation functional (Beck, 1993). The differences between PCM and COSMO implicit solvent models consists in the particular way in which the cavity containing the studied system is created. The specific choice of the implicit solvent model in the performed calculations was conditioned by the capabilities of the used software tool.

## Results and discussion

**FIGURE 2:**
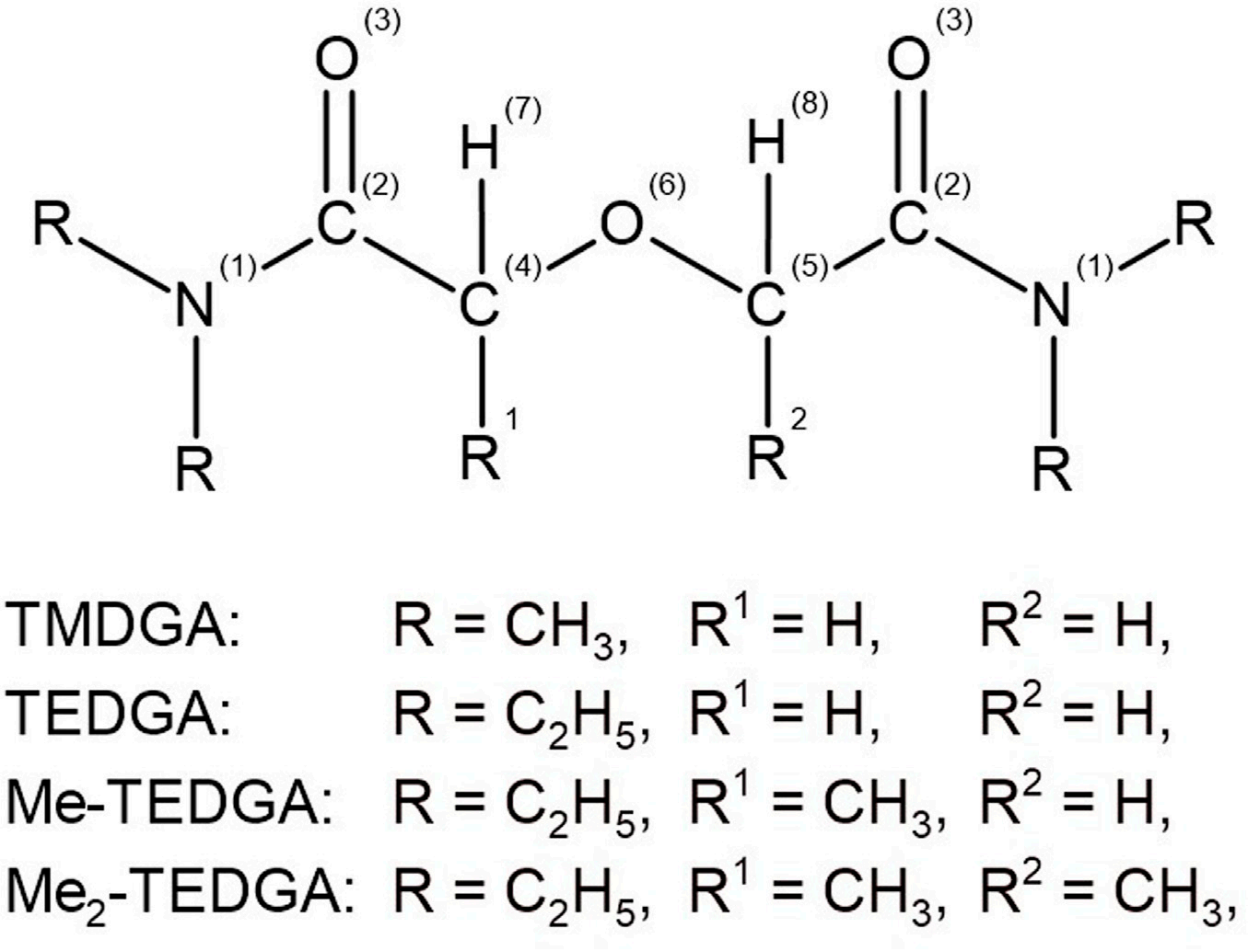
General chemical structure of the studied DGA derivatives with the atom labels indicated.

At first, the geometrical optimization of the studied acid model systems was performed, leading to optimized conformations, indicating a localized direct interaction of the acid representatives with the extractant molecule, mediated by hydrogen atoms. These optimized conformation were then used in subsequent calculations of the studied stability indicators. The atomic denotation used in the calculations and discussion of the results achieved for the studied structures is shown in Figure 2.

As in the previous work (Koubský and Luštinec, 2018), arithmetic averages are used for the symmetrically equivalent atoms to simplify the stability indicators analysis and also to reduce the conformation dependence of the results. Structure of Me-TEDGA is unsymmetrical due to the methyl group bonded on one of the ether carbons C (4,5). For this reason, the atoms C (4,5) are considered as inequivalent; the remaining atoms symmetrical against oxygen O (6) are analyzed as being equivalent ones.

### Radical Fukui function

**FIGURE 3:**
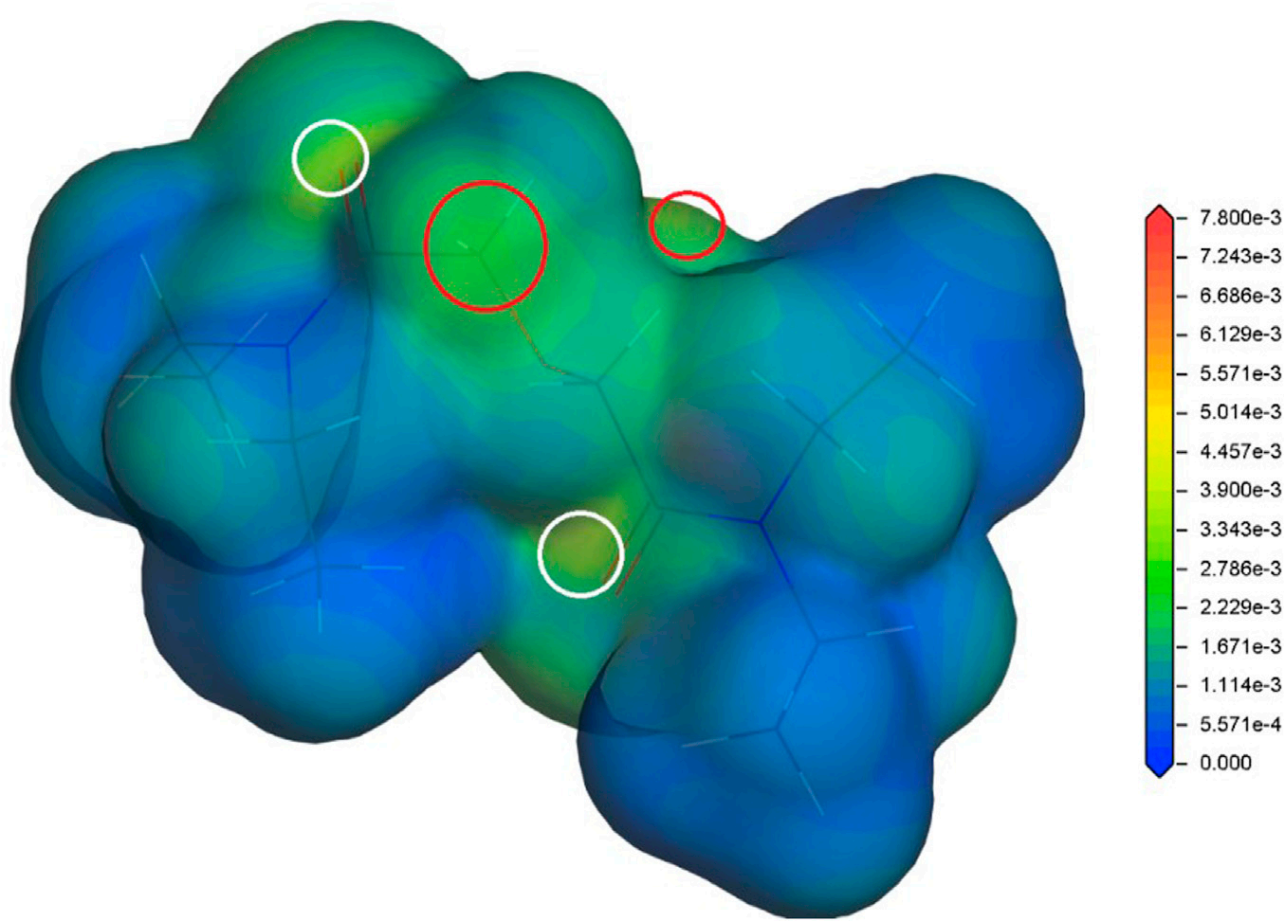
Radical Fukui function for TEDGA/acid-free model (Koubský and Luštinec, 2018), mapped on the electron density iso-surface 0.017 eÅ^−3^; the red circles mark the FF maxima on hydrogens adjacent to ether group, the white circles identify maxima on amide groups (Dmol^3^, DNP, B3LYP).

**FIGURE 4:**
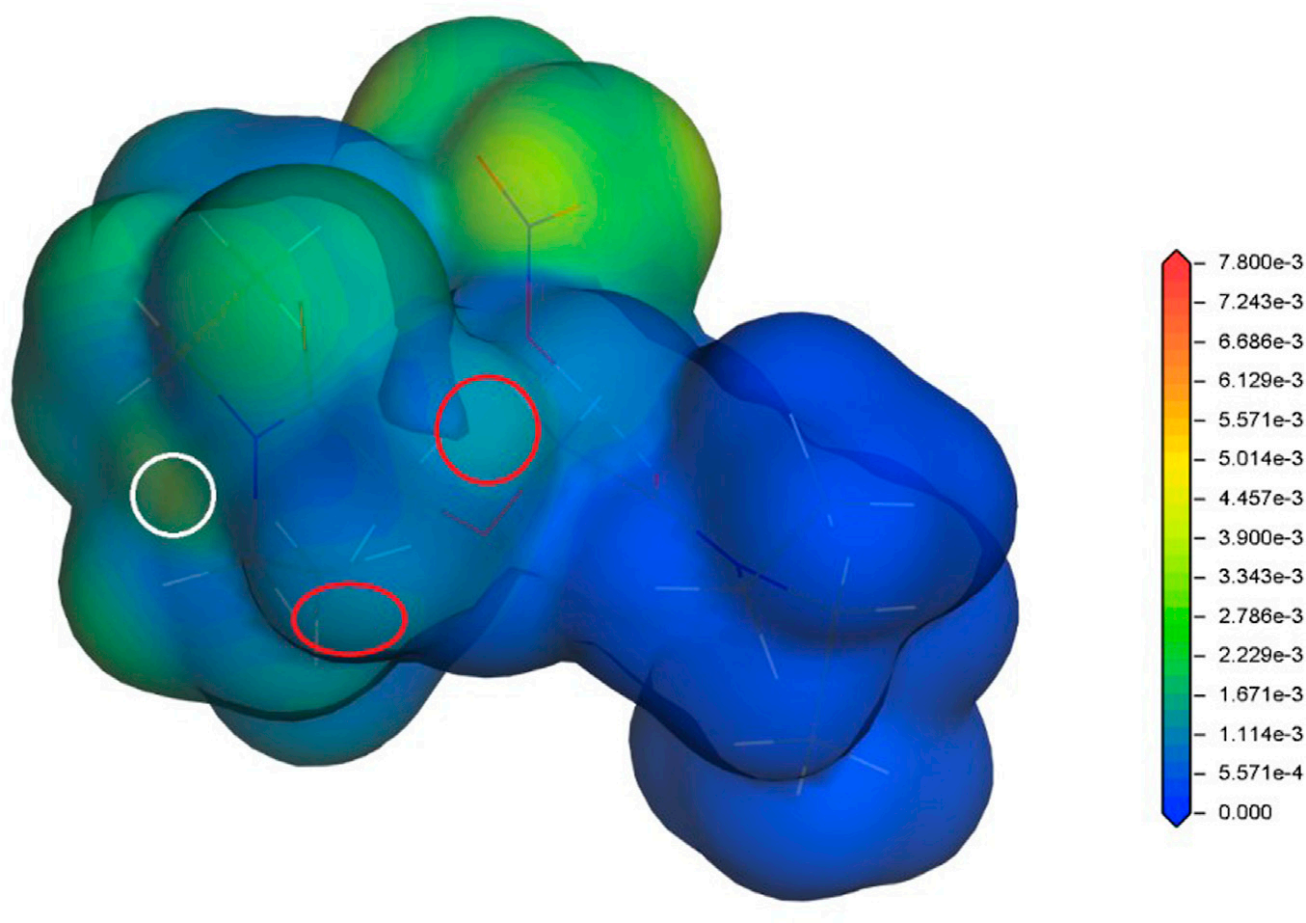
Radical Fukui function obtained for TEDGA/HNO3 acid model, mapped on the electron density iso-surface 0.017 eÅ^−3^; the red circles mark the FF maxima on hydrogens adjacent to ether group, the white circles identify the maxima on amide groups (Dmol^3^, DNP, B3LYP).

In our previous studies (Koubský et al., 2017; Koubský and Luštinec, 2018), the radical Fukui function (FF) (Yang and Parr, 1985) has proved to be a radical stability descriptor relevant for the investigated hydrophilic and lipophilic DGA derivatives. Therefore, the radical FF is also evaluated for the proposed DGAs acid models. The calculated values of radical FF are mapped on electron density iso-surface, and the maxima identified. Similar to the previous study (Koubský and Luštinec, 2018), the main maxima appear in a close vicinity of the ether hydrogens and the amide groups. This trend is found for all tested combinations of DGA derivatives and acid representations. The results obtained for TEDGA are given in Figure 3 and Figure 4 as examples.

The trend of radical FF maxima located in vicinity of ether hydrogens (Table 1) agrees well with the expectations based on experimentally observed radiolytic stabilities of the derivatives. For all acid models, the calculated values of radical FF maxima decrease with the molecular weight of the derivative growing, the trend being even more pronounced than in the case of pure water environment considered in (Koubský and Luštinec, 2018). Within the three acid models considered here, the mentioned trend seems to be stronger for the H^+^ and H3O^+^ models than for the HNO_3_ one. However, as already stated, a direct quantitative comparison of the radical FF values obtained for the different acid models is not straightforward due to the different total charge present in the model systems.

**TABLE 1:** Values of the radical Fukui function in 10^−3^ eÅ^−3^ close to the ether-neighboring hydrogen atoms (red circles in Figure 3 and Figure 4); results for the acid-free model taken from (Koubský and Luštinec, 2018) (DMol^3^, B3LYP, COSMO).

Acid model	TMDGA	TEDGA	Me-TEDGA	Me_2_-TEDGA
Acid-free	3.613	3.613	3.325	3.038
H^+^ model	5.847	4.716	4.235	2.663
H_3_O^+^ model	5.287	4.516	2.463	2.053
HNO_3_ model	2.053	1.322	1.282	1.232

Similar to the acid-free situation (Koubský and Luštinec, 2018), the maxima of radical FF on the amide groups are also observed for all the tested acid models (Table 2). The values obtained for the HNO_3_ acid model are reduced as a whole compared to the acid-free model suggesting that the presence of nitric acid molecule decreases the ability of DGA derivatives to react with radicals. The values obtained for different DGA derivatives show then a gradual descend with the molecular weight increasing, supporting thus the experimentally observed stability trend. For the H^+^ and H_3_O^+^ models, the radical FF values on amide groups fluctuate and are dependent on the location of bonding model species. Also, the influence of different total charge of the model systems must be taken into account.

**TABLE 2:** Values of the radical Fukui function in 10^−3^ eÅ^−3^ close to one of the amide groups (white circles in Figure 3 and Figure 4); results for the acid-free model taken from (Koubský and Luštinec, 2018) (DMol^3^, B3LYP, COSMO)

Acid model	TMDGA	TEDGA	Me-TEDGA	Me_2_-TEDGA
Acid-free	5.284	5.284	5.787	5.787
H^+^ model	7.548	5.787	7.045	6.542
H_3_O^+^ model	5.284	5.032	7.800	5.535
HNO_3_ model	4.781	3.774	3.019	3.019

### Radical Fukui charges and CDD

In order to simplify the analysis and discussion of the calculated results, the volumetric radical FF can be assigned to individual atoms; the obtained condensed values are called atomic Fukui charges. As Table 3 shows, inclusion of acid does not significantly modify the trends found for the acid-free situation (Koubský and Luštinec, 2018). Since also the main features of results obtained with all tested acid models are similar, we discuss them on the example of H3O^+^ acid model. (Although some minor differences can be identified in the case of H^+^ acid model, the arguments remain the same.) The HNO_3_ acid model shows then a significant reduction of all calculated atomic Fukui charges (similar to the situation observed for the radical FF values, *cf.* Table 1 and Table 2) making analysis and a straightforward quantitative comparison with the other two models difficult.

**TABLE 3:** Atomic radical Fukui charges based on Hirshfeld population analysis obtained for studied DGA derivatives and proposed acid models; results for the acid-free model taken from (Koubský and Luštinec, 2018); values for H^8^, H^(R1)^ and H^(R2)^ are identical to the value obtained for H^7^ where relevant (DMol^3^, B3LYP, COSMO).

Acid model	Ligand	N^(1)^	C^(2)^	O^(3)^	C^(4)^	C^(5)^	O^(6)^	H^(7)^
Acid-free	TMDGA	0.062	0.067	0.114	0.030	Eq. C^(4)^	0.037	0.026
	TEDGA	0.057	0.063	0.111	0.030	Eq. C^(4)^	0.033	0.025
	Me-TEDGA	0.057	0.064	0.113	0.021	0.029	0.033	0.023
	Me_2_-TEDGA	0.054	0.064	0.111	0.022	Eq. C^(4)^	0.036	0.020
H^+^ model	TMDGA	0.096	0.071	0.097	0.020	Eq. C^(4)^	0.023	0.025
	TEDGA	0.072	0.068	0.074	0.019	Eq. C^(4)^	0.021	0.024
	Me-TEDGA	0.049	0.076	0.108	0.013	0.018	0.012	0.022
	Me_2_-TEDGA	0.070	0.065	0.088	0.009	Eq. C^(4)^	0.012	0.014
H_3_O^+^ model	TMDGA	0.065	0.072	0.079	0.025	Eq. C^(4)^	0.033	0.031
	TEDGA	0.061	0.070	0.078	0.025	Eq. C^(4)^	0.032	0.029
	Me-TEDGA	0.062	0.068	0.078	0.018	0.020	0.029	0.019
	Me_2_-TEDGA	0.060	0.064	0.083	0.015	Eq. C^(4)^	0.022	0.015
HNO_3_ model	TMDGA	0.038	0.019	0.067	0.009	Eq. C^(4)^	0.022	0.011
	TEDGA	0.045	0.018	0.058	0.006	Eq. C^(4)^	0.007	0.008
	Me-TEDGA	0.044	0.016	0.058	0.002	0.006	0.004	0.006
	Me_2_-TEDGA	0.065	0.018	0.058	0.007	Eq. C^(4)^	0.007	0.011

Due to its high structural fragility, the ether group is the weakest part of DGA molecules when a radical attack is considered. In case of acid-free environment (Koubský and Luštinec, 2018), the values of radical Fukui charges located on the ether group (C^(4,5)^, O^(6)^, H^(7)^, H^(8)^, R^1^ and *R* ^2^ chains) support with the greater (C^(4,5)^, H^(7)^) or lesser (O^(6)^) extend the experimentally observed stability trend. With acid included, the analogical behaviour is observed for C^(4,5)^, H^(7)^ and the equivalent hydrogen atoms. For the ether oxygen O^(6)^, the tendency of atomic Fukui charge to decrease with the ligand molecular weight is remarkably enhanced, suggesting a possible positive influence of acid presence on the tested DGAs derivatives stabilization.

The maxima of radical FF on amide group are also reproduced in the radical Fukui charges located on atoms N^(1)^, C^(2)^, and O^(3)^. Again, the same trends are observed as for the ether group in case of the acid-free model (Koubský and Luštinec, 2018), and remain qualitatively unchanged after the inclusion of acid. The values of charges calculated with the HNO_3_ model are all reduced compared to the acid-free results.

In order to get a deeper insight into the possible reaction mechanism, the CDD indicator values are calculated and evaluated. The calculated results are summarized in (Table 4).

**TABLE 4:** Values of atomic CDD based on Hirshfeld population analysis calculated for the studied DGA derivatives and proposed acid models; results for the acid-free model taken from (Koubský and Luštinec, 2018); values for H^8^, H^(R1)^ and H^(R2)^ equal to the value obtained for H^7^ where relevant (DMol^3^, B3LYP, COSMO).

Acid model	Ligand	N^(1)^	C^(2)^	O^(3)^	C^(4)^	C^(5)^	O^(6)^	H^(7)^
Acid-free	TMDGA	−0.040	0.057	−0.025	0.031	Eq. C^(4)^	0.021	0.018
	TEDGA	−0.044	0.057	−0.020	0.033	Eq. C^(4)^	0.016	0.017
	Me-TEDGA	−0.036	0.059	−0.018	0.017	0.036	0.015	0.014
	Me_2_-TEDGA	−0.033	0.063	−0.017	0.016	Eq. C^(4)^	−0.017	0.006
H^+^ model	TMDGA	−0.034	0.059	−0.040	0.008	Eq. C^(4)^	−0.021	0.015
	TEDGA	−0.033	0.073	−0.031	0.014	Eq. C^(4)^	−0.004	0.018
	Me-TEDGA	−0.066	0.083	0.042	0.031	−0.008	0.001	0.012
	Me_2_-TEDGA	−0.031	0.068	−0.045	0.012	Eq. C^(4)^	0.011	0.010
H_3_O^+^ model	TMDGA	−0.033	0.086	0.014	0.016	Eq. C^(4)^	−0.063	0.015
	TEDGA	−0.035	0.078	−0.003	0.014	Eq. C^(4)^	−0.040	0.011
	Me-TEDGA	−0.035	0.067	−0.006	0.023	−0.001	−0.009	0.000
	Me_2_-TEDGA	−0.028	0.079	0.044	0.011	Eq. C^(4)^	−0.012	0.003
HNO_3_ model	TMDGA	−0.059	−0.031	−0.120	−0.015	Eq. C^(4)^	-0.044	−0.017
	TEDGA	−0.084	−0.035	−0.106	−0.008	Eq. C^(4)^	−0.013	−0.011
	Me-TEDGA	−0.084	−0.029	−0.109	−0.009	0.000	0.000	−0.009
	Me_2_-TEDGA	−0.082	−0.027	−0.101	−0.006	Eq. C^(4)^	−0.020	−0.017

In general, for the acid-free model, the CDD absolute values on almost all atoms show a decreasing trend with the ligand weight growing, conformal with the experimental stability trend. The signs then indicate that atoms N^(1)^ and O^(3)^ are likely the only ones that are susceptible to an electrophilic attack. The remaining atoms might be vulnerable to a nucleophilic attack. This result supports the reaction degradation mechanism proposed by Wilden et al. (2018) based on the electron transfer from the amide group. In addition, in the case of H_3_O^+^ acid model, the position O^(6)^ is predicted to be sensitive to an electrophilic attack. The similar behaviour is observed for TMDGA and TEDGA in frame of the H^+^ acid model, for Me_2_-TEDGA in the acid-free model, and for all tested derivatives in the case of the HNO_3_ acid model. Thus, apparently, selection of a particular acid model affects significantly the afterward obtained CDD values.

### Bond orders

For all tested ligands and acid models, the order of C^(4)^-O^(6)^ bond is found to be the lowest one of all bonds. The calculated C^(4)^-O^(6)^ bond order values are summarized in Table 5. Similar character of the C^(4)^-O^(6)^ bond was also identified for the lipophilic DGA derivatives (Koubský et al., 2017). For all tested models (with and without acid), presence of the methyl group(s) taking place in Me-TEDGA and Me_2_-TEDGA derivatives causes then a small drop in C^(4)^-O^(6)^ bond order. Again, such effect is also observed for the analogically modified lipophilic DGA representatives (Koubský et al., 2017).

**TABLE 5:** Calculated bond order (Wiberg bond indices) of the C^(4)^-O^(6)^ and equivalent C^(5)^-O^(6)^ bond; results for the acid-free model taken from (Koubský and Luštinec, 2018) (Gaussian,B3LYP,PCM,NBO).

Acid model	Bond	TMDGA	TEDGA	Me-TEDGA	Me_2_-TEDGA
Acid-free	C^(4)^-O^(6)^	0.904	0.904	0.888	0.884
	C^(5)^-O^(6)^	Eq. to C^(4)^-O^(6)^	Eq. to C^(4)^-O^(6)^	0.900	Eq. to C^(4)^-O^(6)^
H^+^ model	C^(4)^-O^(6)^	0.895	0.908	0.895	0.880
	C^(5)^-O^(6)^	Eq. to C^(4)^-O^(6)^	Eq. to C^(4)^-O^(6)^	0.913	Eq. to C^(4)^-O^(6)^
H_3_O^+^ model	C^(4)^-O^(6)^	0.913	0.914	0.892	0.895
	C^(5)^-O^(6)^	Eq. to C^(4)^-O^(6)^	Eq. to C^(4)^-O^(6)^	0.914	Eq. to C^(4)^-O^(6)^
HNO_3_ model	C^(4)^-O^(6)^	0.904	0.903	0.880	0.882
	C^(5)^-O^(6)^	Eq. to C^(4)^-O^(6)^	Eq. to C^(4)^-O^(6)^	0.904	Eq. to C^(4)^-O^(6)^

Bond orders closely relate to the other frequently used partitioning quantity of electron density - atomic partial charges. However, the partial charges obtained with the here tested acid models are found to be strongly dependent on the particular total charge included in the model, and show also a high sensitivity to the particular position in the ligand where the acid-representing group is attached (as a result of the geometrical optimization of the system); no reliable interpretation of the partial charges thus can be achieved and their values are not discussed here.

## Conclusion

Three simplified models of acid influence on the radiolytic stability of hydrophilic DGA representatives are proposed and applied in calculations of the selected chemical stability indicators: radical FF, radical Fukui charges, CDD, and bond orders. The results obtained for the individual acid models are compared and juxtaposed with the results obtained for models with no acid influence considered. The newly tested CDD indicator shows absolute values that are generally in agreement with the experimentally observed radiolytic stability trend (TMDGA < TEDGA < Me-TEDGA < Me_2_-TEDGA (Wilden et al., 2018)). For different acid models however, the CDD signs significantly vary, and the CCD indicator fails to provide a reliable stability description.

All the tested acid models provide results similar to the results of the acid-free model, with the main trends remaining unaffected. However, values of the tested indicators as a whole drop down with the acid models applied, suggesting that in addition to the direct protection reflected by the local variations of calculated indicators, some indirect protection mechanism may also originate from acid presence, originating in a general decrease in the chemical reactivity of the ligands in the presence of acid.

Arguments for a direct acid protection effects follows from the two following obtained results: 1) the faster stabilizing reduction of the atomic Fukui charge values at the weakest atomic site of the tested DGA derivatives–the ether oxygen atom - found with the all acid models, and 2) the decrease of radical FF maxima on ether hydrogens and amide groups encountered with the HNO_3_ acid model. The latter effect indicates that undissociated acid molecules may reduce the reactivity of the studied DGA derivatives with radicals, the final protection effect being then dependent on the specific nitric acid concentration applied.

Considering the similarity between the basic trends of radical FF and Fukui charges found for the acid models and the acid-free model, the obtained results in any case do not contradict the proposition made by Horne and co-workers (Wilden et al., 2018) that the significant indirect acid protection effect consists in the preferential reaction of acid with the products of solvent radiolysis, decreasing thus the subsequent direct radical attack rate of the solvent radiolysis products on the extractant molecules.

## Data Availability

The original contributions presented in the study are included in the article/supplementary material, further inquiries can be directed to the corresponding author.
